# Clinical and pulmonary function changes in cough variant asthma with small airway disease

**DOI:** 10.1186/s13223-019-0354-1

**Published:** 2019-07-02

**Authors:** Honglei Yuan, Xiaojing Liu, Li Li, Gang Wang, Chunfang Liu, Yuzhen Zeng, Ruolin Mao, Chunling Du, Zhihong Chen

**Affiliations:** 1 Respiratory Division of Zhongshan Hospital, Shanghai Institute of Respiratory Disease, Fudan University, No. 180 Fenglin Road, Shanghai, China; 2 Respiratory Division of the Affiliated Hospital of Qingdao University, Qingdao, China; 3 Department of Respiratory and Critical Care Medicine, Clinical Research Center for Respiratory Disease, West China Hospital, Sichuan University, Chengdu, China; 4 Department of Laboratory Medicine, Huashan Hospital, Shanghai Medical College, Fudan University, Shanghai, China; 5 Respiratory Division of Qingpu Hospital Affiliated to Zhongshan Hospital, Fudan University, Shanghai, China

**Keywords:** Cough variant asthma, Small airway disease, Forced expiratory flow at 50% (FEF_50%_)

## Abstract

**Background:**

It is known that small airway disease is present across all asthma severities; however, its prevalence and clinical characteristics in cough variant asthma (CVA) have not been fully illuminated.

**Methods:**

A total of 77 CVA patients with preserved proximal airway function (FEV1/FVC > 70%) were enrolled in this study. The correlation between forced expiratory flow at 50% (FEF_50%_) and FEF_25–75%_ in the CVA population was first evaluated. FEF_50%_ was determined to be an easy and feasible parameter for identifying small airway disease. CVA with small airway disease is defined as FEF_50%_ < 70%, whereas CVA with normal small airways is identified as FEF_50%_ > 70%. Demographic features, clinical characteristics, lung function and induced sputum test results were determined at the initial visit and at the final visit 1 year later.

**Results:**

FEF_50%_ is a good marker for small airway disease. The cutoff value of 70% is more sensitive than the previously published 60% for identifying more patients with small airway problems early. Nearly half of the CVA population (45.4%) in our cohort had small airway disease. In both group, symptoms improved greatly after anti-asthmatic treatment. Interestingly, the changes in symptom scores [Asthma Control Test (ACT) and ACQ] were even greater in the CVA with small airway disease group than in the control group because of the higher medication usage in this subpopulation in real life. However anti-asthmatic therapy can not reverse small airway dysfunction. At last visit, FEF_50%_ of CVA with small airway diseases was 57.2% ± 10.5%, still much lower than the control group (FEF_50%_ = 92.6% ± 16.5%).

**Conclusions:**

In our cohort, nearly half of the CVA population had small airway disease. Their demographic features, clinical characteristics, airway eosinophils and drug responsiveness were quite similar between two groups, which means these indices can not be used as markers to identify small airway obstruction. We found FEF_50%_ is an easy and feasible marker for early identification. Regular anti-asthmatic medication helped to improve clinical scores in patients with small airway disease, but the obstruction could not be reversed over 1-year period.

## Background

The small airways are those with an internal diameter less than 2 mm. They extend from the 8th generation airways to the alveoli. The total cross-sectional surface area of small airways is much greater than that of large airways; however, it only contributes to 10–29% of total airway resistance [1, 2]. That is why small airways are referred to as a “silent zone” where chronic disease can accumulate over many years without being noticed [3, 4].

Recently, increasing evidence indicates that inflammatory infiltration and functional impairment affect not only large airways but also small airways in asthmatics [5, 6]. In a systematic review, Usmani et al. [7] examined 15 studies of small airway diseases in adult asthma; they found that the overall prevalence of small airway disease was approximately 50–60% and that it was present across all asthma severities. In severe asthma, Berge et al. [8] found that inflammatory processes and mucus plugging were present in both large and small airways. Furthermore, high-resolution computerized tomography (HRCT) scanning has shown that small airway disease is also present in milder asthma, likely because of greater air trapping in mild disease.

Cough variant asthma (CVA) is a distinct asthma subset in which the only respiratory symptom is chronic cough. It shares the pathophysiological features of bronchial hyperresponsiveness and eosinophilic infiltration with classic asthma but is relatively milder in severity [9]. In this study, the proportion of small airway disease in CVA and its clinical characteristics, treatment regimen and lung function outcomes were observed for a year. Forced expiratory flow at 50% (FEF_50%_) was used as a measurement of early small airway disease in our study. The correlation between FEF_50%_ and FEF_25–75%_ was also compared and discussed.

## Methods

### Study population

The participants included in this study were enrolled in the electronic medical databases (EMD) of the Respiratory Division of Zhongshan Hospital, Huashan Hospital, Qingpu Branch of Zhongshan Hospital in Shanghai. A total of 250 subjects were screened, and 173 subjects were excluded due to wrong phone number, unwillingness to participate, smoking or age limitations. Finally, 77 diagnosed CVA patients were enrolled. Inclusion criteria: (1) diagnosed with CVA, (2) 18–70 years of age, (3) FEV1/forced vital capacity (FVC) > 70%, forced expiratory volume in the first second (FEV1) of at least 80% of the predicted value (FEV1/FVC > 70%, and FEV1% > 80%), (4) all subjects have less than 12% improvement in FEV1 after bronchodilator inhalation. Exclusion criteria: (1) asthma exacerbation within the previous 3 months, (2) FEV1/FVC < 70%, (3) FEV1/FVC > 70% but FEV1% < 80%, (4) upper respiratory infection within the previous 3 months, (5) severe organ dysfunction (for example, respiratory failure, heart failure, liver or kidney failure), (6) pregnancy, (7) unwillingness to be followed for 1 year, (8) smoker. All participants were diagnosed with CVA according to the definition made by ACCP which include: (1) patients present with cough in isolation; (2) patients with normal routine spirometry and bronchial challenge testing reveals the presence of bronchial hyperresponsiveness; (3) the resolution of cough due to specific antiasthmatic treatment [10].

The participants were interviewed in person at the Respiratory Division of Zhongshan Hospital using a structured questionnaire to obtain information about symptoms, lifestyle, medical history, etc. (Table 1). An asthma history questionnaire inquiring about the chief complaint, cough features, sneezing, runny nose in cold air, sputum properties, family history, allergens, and smoking history was completed at baseline. All the participants provided written informed consent. The protocol (No: B2014-109) was approved by the institutional review board at Fudan University prior to the study.

**Table 1:** Characteristics of the study population at baseline

	CVA with low FEF_50%_ (n = 27)	CVA with normal FEF_50%_ (n = 30)	p value
Age (years)	43.2 ± 14.1	41.1 ± 15.6	0.33
Gender (male: female)	56%/44%	27%/73%	0.48
Medical history			
Daytime cough	11 (40.7%)	6 (20%)	0.33
Nighttime cough	19 (70.4%)	23 (77.6%)	0.68
Cough affects sleep	19 (70.4%)	15 (50.0%)	0.57
Chest tightness	20 (74.1%)	10 (33.3%)	0.40
Allergic rhinitis	17 (62.9%)	22 (73.3%)	0.15
Allergic family history	15 (55.6%)	15 (50.0%)	0.27
Pulmonary function test			
FEV1/FVC (%)	77.4 ± 6.06	86.0 ± 6.12	< 0.001
FEV1%	90.77 ± 11.4	99.8 ± 13.6	0.97
FEF_50%_	62.95 ± 8.06	85.7 ± 16.7	< 0.001
Asthma control assessments			
ACT	17.9 ± 4.4	18.3 ± 3.0	0.57
ACQ	1.79 ± 0.99	1.43 ± 0.66	0.14
Induced sputum			
Total number of cells (10^5^/mL)	5.6 ± 1.2	6.7 ± 0.9	0.45
Neutrophils (%)	30.5 ± 6.2	47.2 ± 10.9	0.67
Eosinophils (%)	3.6 ± 0.2	2.7 ± 0.4	0.55
Lymphocytes (%)	7.8 ± 0.6	9.2 ± 0.2	0.23
Macrophages (%)	36.4 ± 4.2	32.3 ± 3.3	0.78

### Asthma control assessments

The Asthma Control Test (ACT) and the Asthma Control Questionnaire (ACQ), designed to assess asthma management, were implemented at baseline (v1 visit) and the follow-up conducted 12 months after baseline (v2 visit). The ACT was rated on a scale of 0–25, where scores of 25 demonstrated complete asthma control, 20–25 signified good asthma control (well-controlled asthma), and scores below 20 indicated that the patient’s disease was not controlled. The ACQ was scored on a scale of 0 to 6 for each question, where 0 represented good control and 6 represented very poor control. There were seven questions in the questionnaire for total score of 42 [11]. The validity and reproducibility of the ACT and ACQ have been described elsewhere [12, 13].

### Pulmonary function test

Spirometry and plethysmographic measurements were performed using standard techniques according to ATS-ERS recommendations. All spirometry was performed by well-trained technicians, and all measurements were repeated three times. Interpretative strategies for lung function tests were previously established and subsequently extended.

### Induced sputum

All participants underwent sputum induction at the first visit. An aerosol of hypertonic saline at a 3% concentration was generated by an ultrasonic nebulizer. Sputum was induced by three consecutive nebulization sessions that lasted 7 min each time. After the sputum bolt was picked out and incubated with DTT, the cells were suspended with PBS. The cell suspension was sent to the lab and stained by hematoxylin–eosin (HE) staining.

### Follow-up

The cohort members were followed for asthma management with in-person follow-up surveys administered 12 months later. The follow-up visit included a history review and physical examination, the ACT/ACQ, and lung function testing. Medications and treatment duration were also recorded for all study participants.

### Statistical analysis

Continuous variables including lung function parameters, ACT/ACQ score, and induced sputum results were tested with a two-sample t-test. The Chi-square test used for classification variables such as sex and age. All statistical analyses were performed using SPSS software, version 21 (IBM Corporation, Somers, NY, USA). All p values were calculated by two-sided tests and were considered statistically significant if p was less than 0.05.

## Results

### FEF_50%_ is a feasible parameter for identifying small airway dysfunction early

**Fig. 1:**
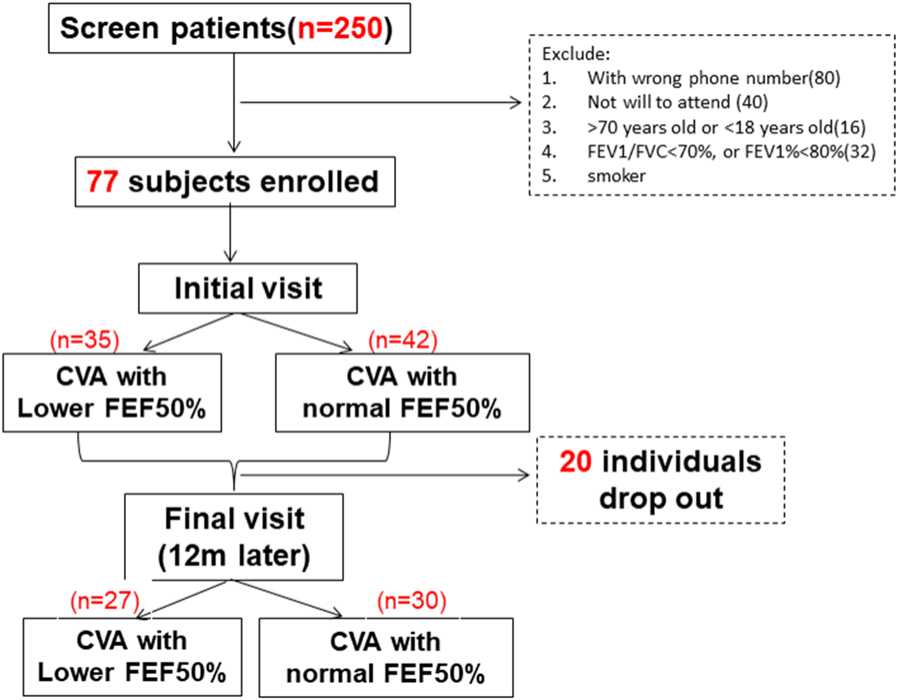
Patients screen and follow up. 250 subjects were screened and 173 subjects were excluded for wrong phone number, no will to attend the trial or age limitation. 77 initially diagnosed CVA patients were enrolled. Participants were divided into two groups: CVA with lower FEF50%, CVA with normal FEF50%. Twenty participants were dropped out during follow up. A total of 57 patients complete the observation over a year

We excluded the total of 173 participants identified through the EMD, in which wrong phone numbers (80 participants), unwillingness to participate (40 participants), age other than 18–70 years (16 participants), patients with proximal airway obstruction (FEV1/FVC < 70% or FEV% < 80%) (32 participants), smoker (5 participants). After these exclusions, a total of 77 participants remained in the study. The participants were divided into two groups according to FEF_50%_, which reflects small airway function: 35 (45.4%) were in the lower FEF_50%_ group (the group with small airway disease) and 42 (54.6%) were in the normal FEF_50%_ group (the group with normal small airways). Twenty participants dropped out during follow-up. Finally, 57 were included in the analysis, with 27 in the lower FEF_50%_ group and 30 in the normal FEF_50%_ group (Fig. 1).

**Fig. 2:**
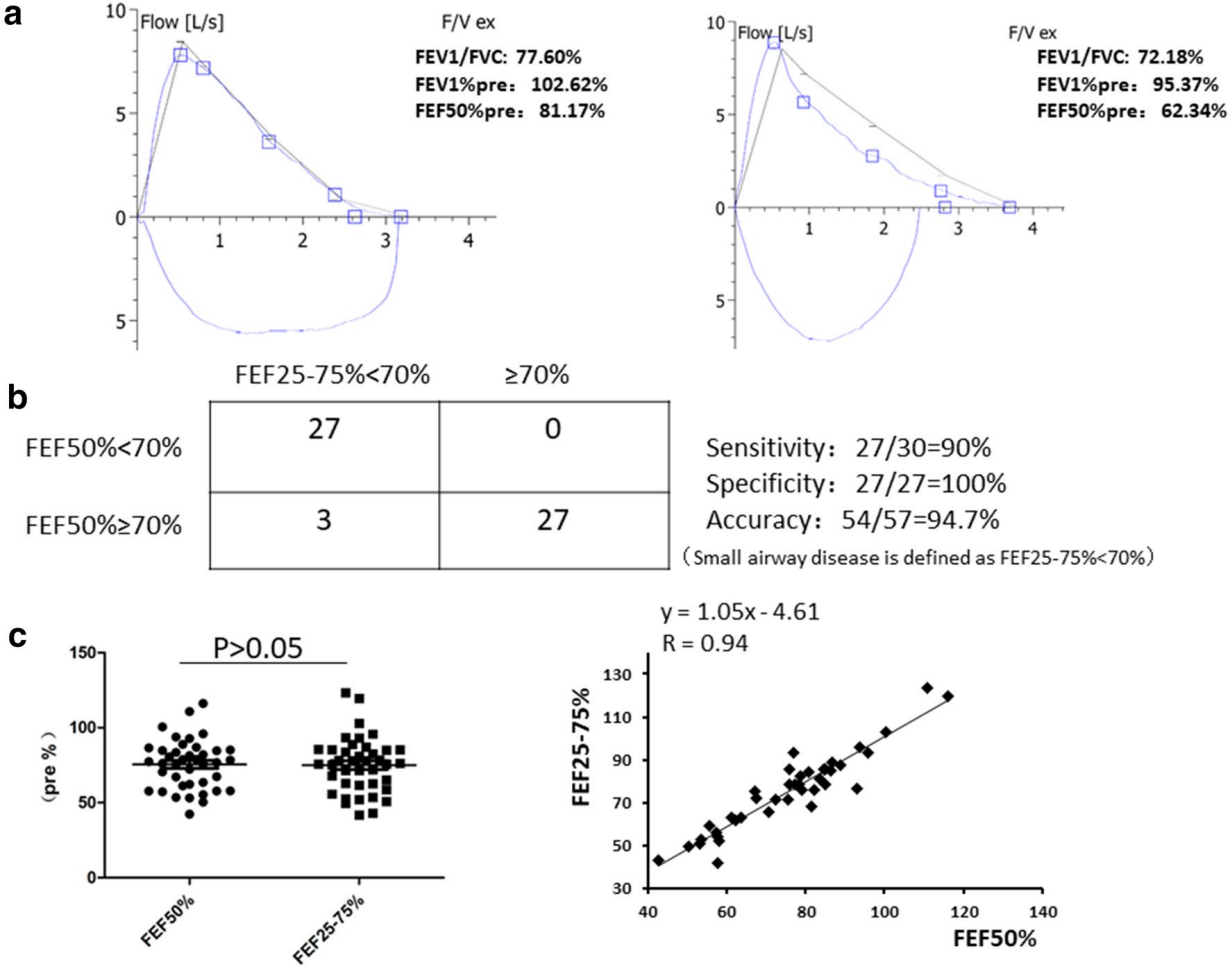
FEF50% and FEF25–75% as early markers of small airway disease. **a** Representative spirometry results in our cohort are showed. Normal airway is the group CVA with normal FEF50%. Small airway disease is the group CVA with lower FEF50%. **b** Diagnostic test of FEF50%. FEF25–75% is defined as gold standard and FEF25–75% < 70% predicted is set as cut value. The sensitivity, specificity and accuracy of new measurement (FEF50%) are measured. **c** The average value of FEF50% and FEF25–75% (p > 0.05). The linear correlation analysis of FEF50% and FEF25–75% in CVA patients. All spirometries were performed by well-trained technician with all data repeated three times

The baseline characteristics of the participants are presented in Table 1. The participants in the lower FEF_50%_ group had lower FEV1/FVC values than those in the normal FEF_50%_ group (Fig. 2a). No differences in symptoms, allergy histories, ACT/ACQ, or induced sputum results between the two groups were observed.

The clinical characteristics, airway eosinophils and drug responsiveness are quite similar in patients with and without small airway disease (Table 1); consequently, more sensitive parameters are urgently needed to identify small airway disease early. FEF_25–75%_ has been shown to be acceptable parameter to evaluate small airway disease [14]. However, it has not been fully accepted by American Thoracic Society (ATS) for determining small airway disease because of its variability compared with FEV1. Thus, we hypothesized that FEF_50%_ may be a substantial parameter with better feasibility. Using FEF_25–75%_ as the ATS old standard, we examined the methodology of FEF_50%_ as a diagnostic test for small airway impairment in CVA patients. We found a sensitivity of 90% and a specificity of 100%. The accuracy was 94.7% (Fig. 2b). The mean values of FEF_50%_ and FEF_25–75%_ were 74.12% ± 2.02% and 75.89% ± 2.1%, respectively (p > 0.05). The correlation coefficient was 0.94. The extremely close mean values and good correlation prompted us to conclude that FEF_50%_ is an acceptable spirometric parameter for distinguishing small airway dysfunction from normal small airway function. Furthermore, the regression equation was y = 1.05x − 4.61 (Fig. 2c).

### Clinical characteristics of CVA with small airway disease over a 1-year period

**Fig. 3:**
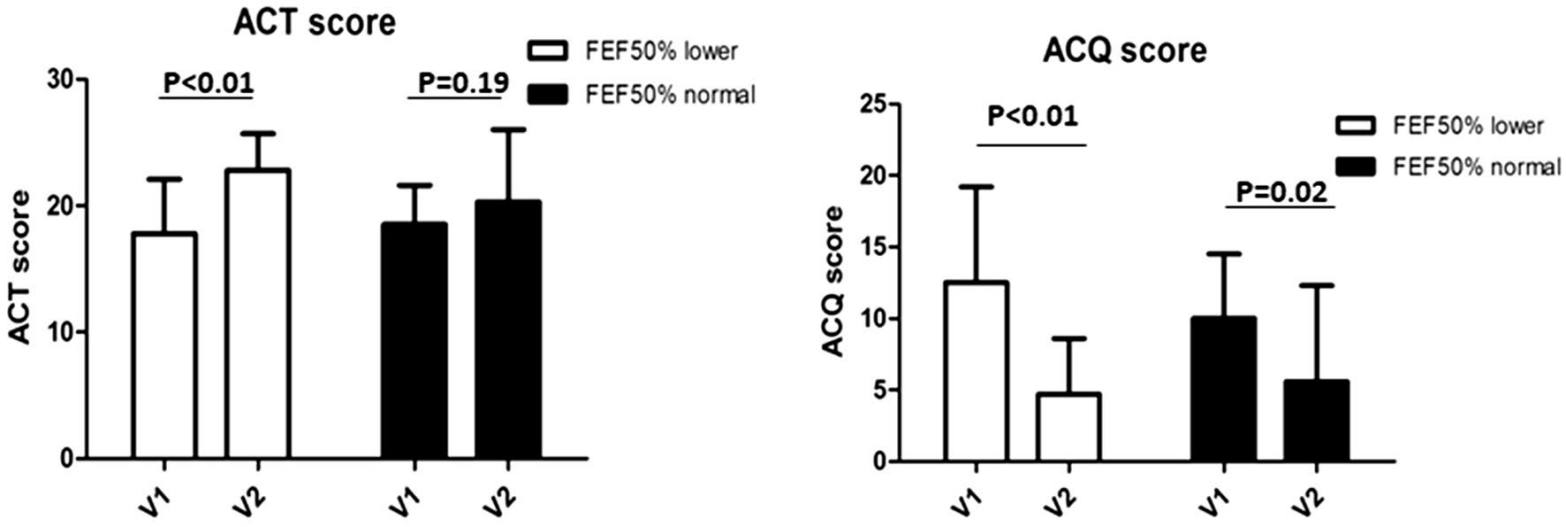
Asthma clinical control over a year by ACT and ACQ questionnaire. Both ACT and ACQ score improved in both groups when compared final visit with initial visit. The scale of improvement in CVA with lower FEF50% is much greater than the control groups

The ACT/ACQ scores showed little difference between the two groups at baseline, indicating that the patients in both groups had similar symptoms and poor control (Table 1). During the follow-up interview, we found that the ACT scores increased significantly in the lower FEF_50%_ group, indicating that CVA improved from not controlled to well controlled (17.9 ± 4.5 to 22.1 ± 2.6). There was a trend of improvement of ACT scores in the normal FEF_50%_ group (18.6 ± 3.2 to 20.4 ± 5.0), however it didn’t reach a statistical significance. The ACQ score decreased in both groups, both of which reach a statistical significance (Fig. 3).

**Fig. 4:**
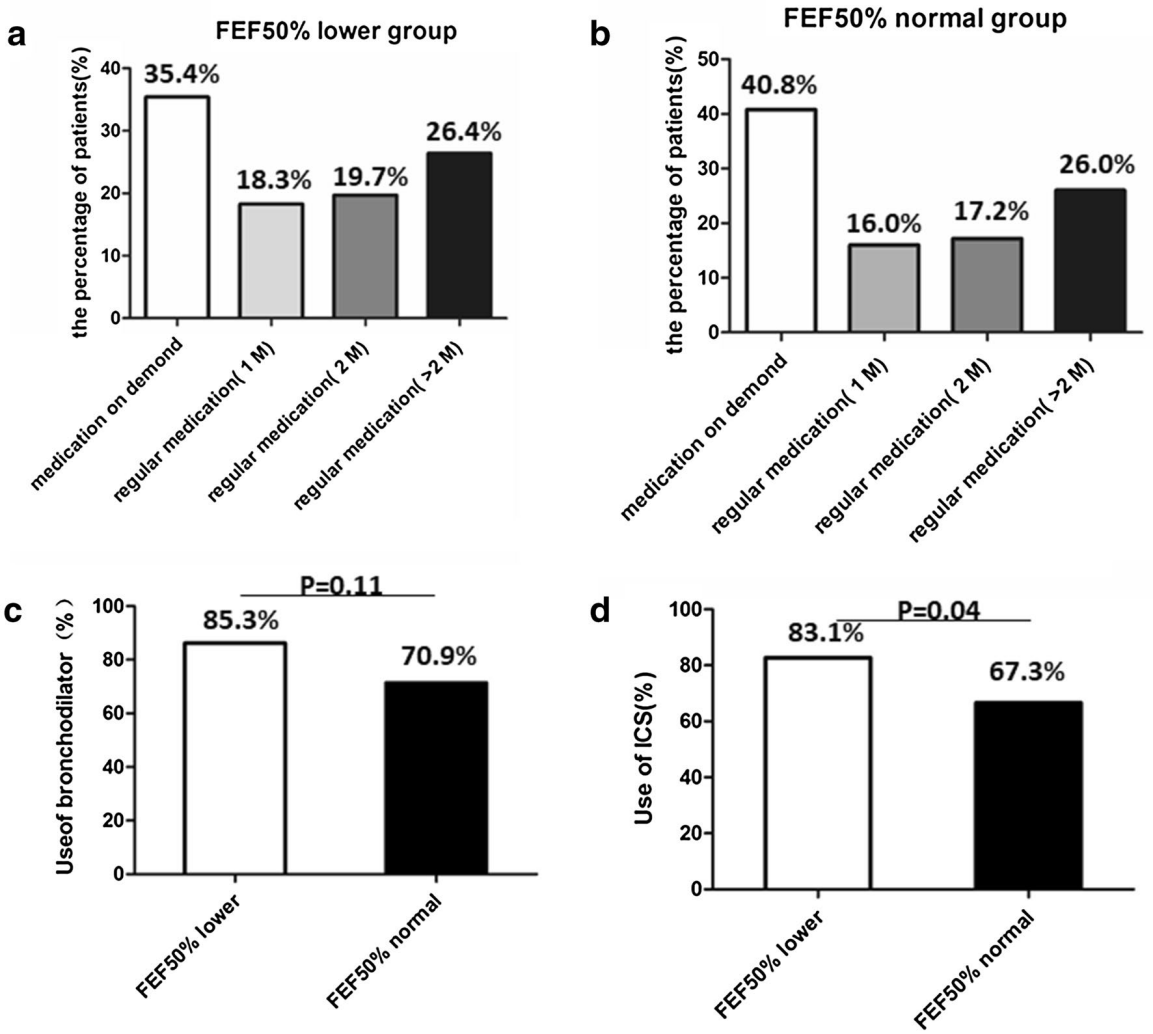
Medication usage for CVA cohort in real-life world. The application timing of medication usage in lower FEF50% group (**a**) and normal FEF50% group (**b**). **c** The rate of bronchodilators usage in the two groups. **d** The rate of ICS usage in the two groups. p < 0.05: statistically significant

**Fig. 5:**
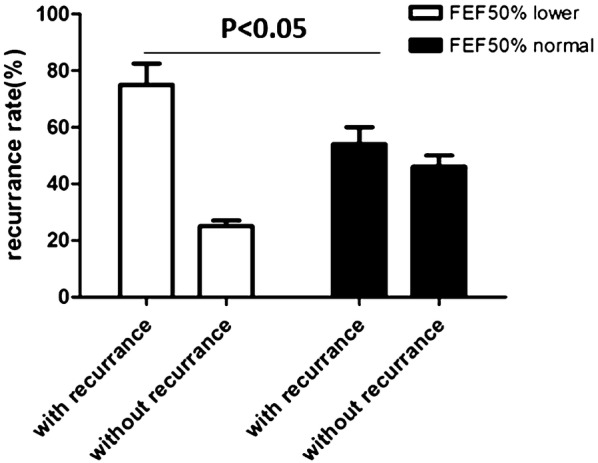
Recurrence of CVA over a year. The recurrence rate in CVA with FEF50% lower group is higher than that of control group (p < 0.05: statistically significant)

The significant improvement of asthma control in the lower FEF_50%_ group prompted us to further investigate medication usage throughout the follow-up period. We found that 64.6% of the patients in the lower FEF_50%_ group insisted on regular medication more than 1 month, while 59.2% in the normal FEF_50%_ group. Obviously, the patients in the lower FEF_50%_ group tended to use bronchodilator and ICS more frequently, especially they used more ICS than control group (83.1% to 67.3%, p = 0.04) (Fig. 4). We presumed “small airway dysfunction” in pulmonary function report prompted patients more adherent to treatment. This may explain the apparent improvement of asthma control in the lower FEF_50%_ group. Then, we examined the overall recurrence in the two groups. Although it can get good clinical control, the recurrence rate in the lower FEF_50%_ group was much higher than that in the normal FEF_50%_ group (− 75% ± 7.6% vs 54% ± 4.9%, p < 0.05) (Fig. 5).

### Lung function changes in CVA with small airway disease over a 1-year period

**Fig. 6:**
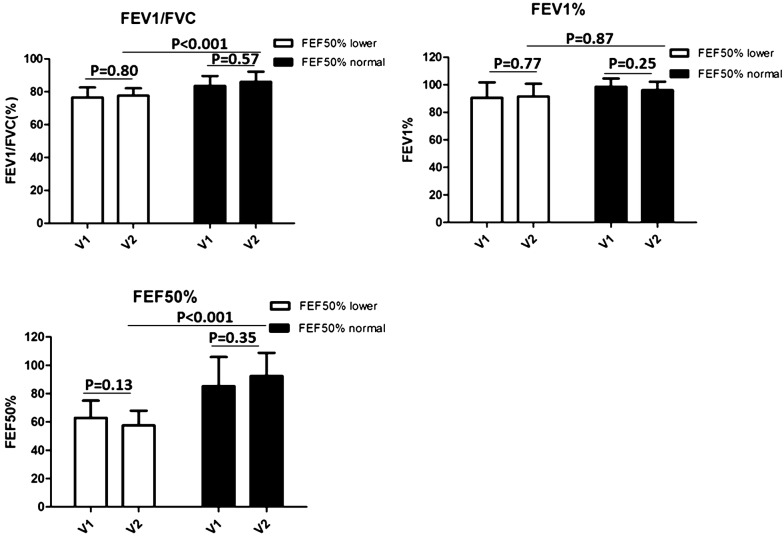
Changes of lung function test between groups over a year. **a** FEV1/FVC at the first visit (V1) and final visit (12 months later) (V2) for lower FEF50% group and normal FEF50% group. **b** FEV1% at the first visit (V1) and final visit (12 months later) (V2) for lower FEF50% group and normal FEF50% group. **c** FEF50% at the first visit (V1) and final visit (12 months later) (V2) for lower FEF50% group and normal FEF50% group. p < 0.05: statistically significant

To investigate the lung function changes in CVA patients with small airway disease, we further compared the parameters of expiratory flow, including FEV1, FEV1/FVC and FEF_50%_. FEV1/FVC and FEV1%, which represent proximal airway function, were slightly improved or almost the same over the 1-year study period (Fig. 6). The same was true of FEF_50%_. When we compared the FEF_50%_ between the two groups at the end of the year, the value was still much lower in the CVA with small airway disease group compared with the group with normal small airways (57.2% ± 10.5% vs 92.6% ± 16.5%). The results indicated that the more regular use of anti-asthma medication could improve clinical symptoms, but not small airway obstruction.

## Discussion

Spirometry is the most frequently used, non-invasive way to assess the airflow limitation in asthma. Parameters such as FEV1 (forced expiratory volume at 1 s) and FEV1/FVC (the ratio of forced expiratory volume at 1 s to forced vital capacity) are widely used to evaluate proximal airway obstruction. The forced mid-expiratory flow between 25 and 75% (FEF_25–75%_) can decrease more steeply than the ratio of FEV1/FVC when airway closure and air trapping occur in the distal airways [15, 16]. Back to 1975, Dosman and colleagues demonstrated FEF_50_ (V_max 50_) reduction is a sensitive measure to distinguish small airway obstruction while breathing helium–oxygen mixture especially in smokers [17]. Also, FEF_25–75%_ has been identified as an early marker of small airway impairment in subjects with allergic rhinitis [14]. Several studies have shown a good correlation between FEF_25–75%_ and the HRCT finding of air trapping. Nevertheless, the cut-point of FEF_25–75%_ for evaluating small airway impairment has been controversial. Marseglia et al. [18] defined the cut-point as < 80% of predicted. Of the 58 subjects with a normal FEV1, FVC, and FEV1/FVC ratio, 20 (34%) had a reduced FEF_25–75%_ with a mean value 70.3 ± 8.5, compared with 108.0 ± 14.3 in the patients with preserved FEF_25–75%_. Manoharan et al. [19] selected a cut-point of 60% of predicted for FEF_25–75%_ to define the presence of small airway disease. A total of 238 patients (54%) had values < 60%.

In our study, the correlation between FEF_50%_ and FEF_25–75%_ in the CVA population was first evaluated. The FEF_50%_ is an instantaneous flow representing the flow rate at half of expiration, whereas the FEF_25–75%_ is an average value over the mid-vital capacity range [20]. When the cut-point of reduced FEF_50%_ was defined as < 60% predicted, 21 out of 57 subjects (36.8%) had a reduced FEF_50%_, and the exact same number and proportion were found when the cut-point of reduced FEF_25–75%_ was defined as < 60%. Whether FEF_50%_ predicted or FEF < 60% predicted were defined as markers of small airway disease, the judgment of the disease was 100% consistent between the two indices (the values of average FEF_50%_ or FEF_25–75%_ were 53.9% and 50.9%, respectively). However, the incidence of small airway disease in our CVA population was relatively low according to this cut-point. We attempted to reset the cut-point of FEF_25–75%_ < 70% as a measure of small airway disease and found that 30 out of 57 subjects (52.6%) had a reduced FEF_25–75%_; 28 subjects’ FEF_50%_ values were also < 70% of predicted, and 2 patients’ values were > 70% of predicted. If FEF_50%_ < 70% predicted or FEF_25–75%_ < 70% predicted are defined as measurements for classifying small airway disease, more patients with early small airway disease will be identified. If the FEF_25–75%_ measurement is defined as ATS old standard, the new indices of FEF_50%_ sensitivity, specificity and accuracy are 90%, 100% and 94.7%. Yuan et al. [21] found that FEF_50%_ was approximately 15% higher than FEF_25–75%_; furthermore, the difference between the two was fairly constant and was well-preserved in cases of irregularly shaped curves. In our study, the value of FEF_50%_ was 6% higher than that of FEF_25–75%_ (58.9% vs 55.6%) if < 70% predicted was used as a measurement of small airway disease, which is consistent with previously published data. Compared with FEF_25–75%_, FEF_50%_ is simple, easy to understand and does not need to be computed.

FEF_25–75%_ has been considered as an index of small airway impairment before. However, the ATS guidelines on lung function testing do not support the use of FEF_25–75%_ to identify small airway disease [22]. FEF_25–75%_ is considered more variable than FEV1 because it is influenced by changes in lung volume and the shape of the flow-volume loop. The weakness is that the value should be varied when a series of spirometry are done in the same individual. In our study, all the CVA patients had preserved proximal lung function (with normal FEV1, FVC and FEV1/FVC). All spirometry was performed by well-trained technicians, and all measurements were repeated three times. Therefore, we suggest that in patients with preserved FEV1, FEF_25–75%_ or FEF_50%_ may be used as marker of early small airway disease.

We used FEF_50%_ < 70% as a marker for small airway disease and found that nearly half of the CVA patients with preserved FEV1 had small airway problems (35/77 = 45.4%), which is similar to the 50–60% prevalence of small airway disease in adult asthma reported by Usmani [7] in a systemic review. It is noteworthy that the clinical symptoms, allergic disease history and inflammation in induced sputum were almost the same between the CVA with lower FEF_50%_ group and the CVA with normal FEF_50%_ group, whereas the FEF_50%_ values differed significantly (62.95% vs 85.7%). In a study of 58 patients with rhinitis, Marseglia et al. [18] demonstrated that the proportion of subjects with reduced FEF_25–75%_ appeared to increase with increasing severity of allergic predictors (rhinitis symptoms, rhinitis eosinophils and BHR). The more severe the allergy score was, the lower the mean FEF_25–75%_ value appeared to be. However, in our CVA cohort, a link between clinical scores, airway inflammation and the FEF_50%_ value was not observed. The underlying pathogenesis of early small airway dysfunction in CVA patients requires further investigation.

Anderson et al. [23] determined the prevalence of small airway disease in populations with differing asthma severities. Denlinger et al. [24] showed that small airway dysfunction, measured by FEF_25–75%_, was positively correlated with exacerbation frequency in both adults and children with exacerbation-prone asthma. Similarly, children from a Boston cohort with reduced FEF_25–75%_ had a substantially higher risk of exacerbations and systemic steroid use compared with children with normal lung function [25]. Our cohort study not only illustrated the prevalence of small airway disease in one subtype of mild asthma—CVA—but also observed the outcome of CVA with small airway disease over a 1-year period. The symptom scores (ACT and ACQ) at follow-up in CVA patients with small airway disease improved markedly compared with the initial visit. Interestingly, the changes in scores (ACT and ACQ) were even greater in the CVA with small airway disease group than in the CVA with normal small airways group. Lung function tests were performed at both the initial and follow-up visits; while parameters of expiratory flow such as FEV1, FEV1/FVC and FEF_50%_ improved slightly or were almost the same after 1 year in both groups, the recurrence rate was significantly higher in the CVA with small airway disease group compared with the CVA with normal small airways group. A team of multinational clinicians initiated a study called the longitudinal assessment of small airways involvement in asthma (ATLANTIS) in 2014. They plan to include 900 subjects and follow them for 1 year. All small airway diseases will be evaluated by spirometry, MBNW, IOS, and CT scan. We await the results of this study to optimize our knowledge of the prevalence and prognosis of asthma with small airway disease [26].

In the cohort, the participants received therapy according to standard clinical care, without any further pharmacological intervention. For the CVA patients with small airway disease, the FEF_50%_ value was almost unchanged after 1 year and was still much lower than the mean FEF_50%_ in CVA patients with normal small airways (57.2% ± 10.5% vs 92.6% ± 16.5%). Interestingly, the asthma control status at the final visit was better in the CVA patients with small airway disease than in those without small airway disease. This is probably due to more regular and longer medication usage in real life among CVA patients with small airway disease. We presume that the notation of “small airway dysfunction” in the lung function report prompted these patients to adhere to treatment and regular check-ups. We found that the ICS-sold (for example budesonide) on the Chinese market did not improve small airway dysfunction over a year. Whether the newly developed extra-fine particle ICS (beclomethasone/formoterol, propelled by hydrofluoroalkane) has the ability to improve small airway disease? It is reported to have a mean aerodynamic diameter of 1–2 μm and can reach small airways, leading to an increase in the lung deposition rate as high as 50–68% [27–29]. Thus, the efficacy of extra-fine particle ICS for CVA patients with small airway disease requires further investigation.

## Conclusion

In conclusion, we found that nearly half of our CVA population had small airway disease. Their demographic features, clinical characteristics, airway eosinophil levels and drug responsiveness were quite similar to those of patients without small airway disease. CVA with small airway disease has higher recurrence rate after anti-asthmatic therapy. FEF_50%_ is suggested as an easy and feasible marker for the early identification of this subpopulation with small airway disease. Unlike FEV1% as a marker of proximal airway obstruction, small airway disease assessments are in their relative infancy, and population-based data are urgently needed. Other techniques, such as MBNW, IOS, alveolar FeNO and micro CT, are also being used to evaluate small airway disease [29–32]. The strengths and shortcomings of different methods will be compared in the future [16, 33, 34]. Longitudinal studies with larger populations are needed to determine whether ultrafine particles affect the outcome of CVA in terms of asthma control, lung function improvement and disease recurrence.

## Data Availability

The datasets used and analysed during the current study are available from the corresponding author on reasonable request.

## Abbreviations

ACQ: Asthma Control Questionnaire
ACT: Asthma Control Test
ATLANTIS: longitudinal assessment of small airways involvement in asthma
ATS: American Thoracic Society
CVA: cough variant asthma
EMD: electronic medical databases
FEV1: forced expiratory volume in 1 s
FEF_50%_: forced expiratory flow at 50%
FEF25–75%: forced mid-expiratory flow between 25 and 75%
FVC: forced vital capacity
HE: hematoxylin–eosin
HRCT: high-resolution computerized tomography
